# Spanish version of the Frontotemporal Dementia Knowledge Scale: adaptation and validation

**DOI:** 10.1590/0004-282X-ANP-2020-0550

**Published:** 2021-12-31

**Authors:** Nahuel Magrath Guimet, Ismael Luis Calandri, Pablo Miguel Bagnati, Matthew Wynn, Ricardo Francisco Allegri

**Affiliations:** Fleni, Clínica de Demencia Frontotemporal, Servicio de Neurología Cognitiva, Neuropsiquiatría y Neuropsicología, Departamento de Neurología, Buenos Aires, Argentina. Fleni, Clínica de Demencia Frontotemporal Servicio de Neurología Cognitiva, Neuropsiquiatría y Neuropsicología Departamento de Neurología Buenos Aires Argentina; 2 Fleni, Servicio de Neurología Cognitiva, Neuropsiquiatría y Neuropsicología, Departamento de Neurología, Buenos Aires, Argentina. Fleni Servicio de Neurología Cognitiva, Neuropsiquiatría y Neuropsicología Departamento de Neurología Buenos Aires Argentina; 3 Global Brain Health Institute, Atlantic Fellow for Equity in Brain Health, University of California, San Francisco United States of America. Global Brain Health Institute Atlantic Fellow for Equity in Brain Health University of California San Francisco United States of America; 4 Washington University in St. Louis, Department of Psychological & Brain Sciences, Saint Louis, United States of America. Washington University in St. Louis Department of Psychological & Brain Sciences Saint Louis United States of America

**Keywords:** Frontotemporal Dementia, Frontotemporal Lobar Degeneration, Aphasia, Dementia, Diagnostic Errors, Neuropsychiatry, Demencia Frontotemporal, Degeneración Lobar Frontotemporal, Afasia, Demencia, Errores Diagnósticos, Neuropsiquiatría

## Abstract

**Background:**

Frontotemporal dementia (FTD) is a neurodegenerative disease and is one of the most common causes of dementia in people under 65. There is often a significant diagnostic delay, as FTD can be confused with other psychiatric conditions. A lack of knowledge regarding FTD by health professionals is one possible cause for this diagnostic confusion.

**Objectives:**

The aim of this study was to adapt and validate the Frontotemporal Dementia Knowledge Scale (FTDKS) in Spanish.

**Methods:**

A translation was done, following cross-cultural adaptation guidelines, which consisted of forward translation, blind back translation, and an analysis by a committee of experts. For the present study, 134 professionals from different health areas responded the Spanish version of the FTDKS. The statistical analysis was performed using R version 4.0.0 “Arbor day” and the Psych, sjPlot packages.

**Results:**

The Spanish version of the FTDKS had good reliability and internal consistency (Cronbach alpha 0.74.). The sample's mean score was 19.78 (range = 4-32, SD 6.3) out of a maximum of 36 points.

**Conclusions:**

The results obtained show that the Spanish version has good psychometric properties. The FTDKS is applicable in our environment and can be a useful tool to evaluate the knowledge of health professionals regarding frontotemporal dementia.

## RESUMEN

**Antecedentes:**

La demencia frontotemporal (DFT) es una enfermedad neurodegenerativa y es una de las causas más comunes de demencia en personas menores de 65 años. A menudo existe un retraso significativo en el diagnóstico, ya que la FTD puede confundirse con otras afecciones psiquiátricas. La falta de conocimientos sobre la DFT por parte de los profesionales de salud es una posible causa de esta confusión diagnóstica.

**Objetivos:**

El presente estudio describe nuestros esfuerzos para adaptar y validar la Escala de Conocimiento de la Demencia Frontotemporal (FTDKS) en español.

**Métodos:**

Se realizó una traducción, siguiendo las pautas de adaptación transcultural, que consistió en una traducción directa, una traducción inversa ciega y un análisis por parte de un comité de expertos. Para el presente estudio, 134 profesionales de diferentes áreas de la salud respondieron la versión en español del FTDKS. El análisis estadístico se realizó utilizando la versión 4.0.0 de R "Arbor day" y los paquetes Psych, sjPlot.

**Resultados:**

La versión en español del FTDKS tiene una buena fiabilidad y consistencia interna (alfa de Cronbach 0,74.). La puntuación media de la muestra fue de 19,78 (rango = 4-32, SD 6,3) sobre un máximo de 36 puntos. **Conclusiones:** Los resultados obtenidos muestran que la versión española tiene buenas propiedades psicométricas. El FTDKS es aplicable en nuestro medio y puede ser una herramienta útil para evaluar los conocimientos de los profesionales sanitarios sobre la demencia frontotemporal.

## INTRODUCTION

Frontotemporal dementia (FTD) is one of the most common causes of dementia in people under 65 years of age, and the third most prevalent cause of dementia altogether^1^. 

Clinically, the FTD syndromes include the behavioral variant of FTD (bvFTD) and two language syndromes, the semantic (svPPA) and the nonfluent/agrammatic (nfvPPA) variants of primary progressive aphasia. Between these FTD syndromes, bvFTD is the most common clinical presentation. It is characterized by personality changes with behavioral disinhibition, apathy, loss of empathy, compulsive or ritualistic behavior, hyperorality, and dysexecutive symptoms^2^. In the language variants, the key component is progressive aphasia.

Unlike other dementias, such as Alzheimer's disease, FTD mainly affects behavior, language, or the motor system. Due to these characteristics, it is often misdiagnosed as a primary psychiatric illness^3^. Importantly, misdiagnosis has a negative impact on patients and their families who seek an answer to symptoms that continue to progress, compromising the patient's personality, isolating them from social ties, undermining the family economy, and further disorienting the professionals who do not know how to deal with this disease. To support proper diagnosis, many workgroups have evaluated potential reasons for the diagnostic delay of FTD^4^^,^^5^^,^^6^. One study evaluated the mean duration from the onset of symptoms to the diagnosis of a neurodegenerative disorder in each of the FTD syndromes (3.7 years for bvFTD, 3.5 years for nfvPPA, and 1.4 for svPPA)^7^. The authors concluded the reasons behind this delay to be related to a misdiagnosis, with the symptoms of FTD being misattributed to a primary psychiatric disorder^3^^,^^4^.

Furthermore, we suspect that diagnosis errors could be even more important in Argentina and the surrounding region. For example, the estimated prevalence of FTD in the US for the population between 45 to 64 years is 15-22 per 100.000^8^. Based on these rates, Argentina (a country with approximately 10,040,258 inhabitants in that age range^9^), should have a prevalence of 1,500 to 2,200 FTD cases. However, Argentina does not maintain an active countrywide registry of these cases, so no reliable epidemiological information regarding FTD exists. Fleni, situated in Buenos Aires, is one of the largest neurology tertiary referral centers in Argentina in which more than 1000 patients with dementia are evaluated annually and clinical care is integrated with extensive research programs. Despite this, Fleni has identified only 50 patients with FTD from its records from 2010 to the present day, likely representing an underestimation. In the literature, it is well recorded that the given prevalence varies from country to country and even in the same country from one study to another^7^^,^^8^^,^^10^^,^^11^^,^^12^^,^^13^. The main reason for this is that this disease is still missed and misdiagnosed and most numbers probably underestimate its true prevalence^4^. However, even if we ignore this fact and accept the estimated cases for this prevalence numbers, the recorded cases in Argentina seem to be below what we would estimate.

Based on these arguments and considering the prevalence and the frequent misdiagnosis, one possible explanation is that health professionals lack important knowledge regarding FTD and thus may fail to diagnose it in its early stages. Given this, it is essential to assess FTD knowledge among health professionals. To accomplish this, Wynn et al. developed the Frontotemporal Dementia Rating Scale (FTDKS). In this 18-item scale, the respondents answer objective questions about FTD using a 4-point Likert scale format (False, Probably False, Probably True, True), with an auxiliary “I don’t know” option.

To understand the low frequency of FTD diagnosis in Argentina, our intention was to assess disease knowledge among health professionals. As a first step, we adapted the FTDKS scale into Spanish and report on its psychometric properties.

## METHODS

### Cross-cultural adaptation process

In order to initiate the adaptation to Spanish and validation of the FTDKS, we first asked for and obtained consent from the original author of the scale (Wynn et al.).

Following established guidelines^14^, adapting the FTDKS to Spanish involved four-steps: the forward translation, the blind back translation, a review by an expert committee, and administration to a validation sample.

*Forward translation*

The first stage in the adaptation process was translating the FTDKS into Spanish. A bilingual experimental psychologist from Argentina, familiar with both cultures, translated the survey into Spanish.

*Blind back translation*

A second independent translator, a clinician with the source language (English) as their mother tongue and who was blind to the original version, translated the scale back into English. This process revealed that the Spanish (translated) and English (original) versions reflected the same content. 

*Expert committee*

A committee composed of a cognitive neurologist, neuropsychiatrist, neuropsychologist, and the two translators who performed the initial translation and backward translation assessed semantic, idiomatic, and conceptual equivalence of the Spanish FTDKS.

*Final version*

The final FTDKS Spanish version (Table 1), like the original version, consists of 18 items where respondents answer factual questions about FTD using a 4-point Likert-type scale format (*False*, *Probably False*, *Probably True*, *True*), with an auxiliary *Don’t Know* option. Respondents receive 2 points for a correct *True* or *False* response, 1 point for a correct *Probably True* or *Probably False* response, and 0 points for an incorrect or *Don’t Know* response.

**Table 1.** Psychometric properties of the scale’s items. The statement column represents the final form of the translated item. In parenthesis, the original version following the correct answer.

Item	Statement	Mean	Standard deviation	Skew	Item difficulty	Item discrimination	Α if deleted
1	La Demencia Frontotemporal (DFT) es una variante de la Enfermedad de Alzheimer (Frontotemporal dementiais a type of Alzheimer disease) (F)	1.66	0.68	-1.77	0.83	0.39	0.72
2	Para la mayoría de las personas con DFT los síntomas aparecen antes de los 65 años de edad (For the majority of people with frontotemporal dementia, symptoms appear before they are 65 years old) (T)	1.25	0.85	-0.5	0.62	0.32	0.72
3	Entre todas las personas con demencia, un 5 a 10% de ellos tiene demencia frontotemporal (Among all people with dementia, 5-10% of them have frontotemporal dementia) (F)	0.31	0.65	1.87	0.16	0.02	0.74
4	Las personas que rondan los treinta años de edad pueden tener demencia frontotemporal (People in theirthirties can developsymptomsof frontotemporal dementia) (T)	0.9	0.84	0.2	0.45	0.21	0.73
5	La pérdida de memoria es un problema mayor en la demencia frontotemporal (Memory loss is a major symptom of frontotemporal dementia) (F)	1.37	0.86	-0.81	0.69	0.32	0.72
6	La demencia frontotemporal puede ser transmitida genéticamente de los padres a los hijos (Frontotemporal dementia can be passed down from parent to child) (T)	0.87	0.84	0.24	0.44	0.30	0.73
7	Dentro de las personas menores de 60 años de edad, la demencia frontotemporal es tan común como la enfermedad de Alzheimer (Among people under 60 y old, frontotemporal dementia is about as common as Alzheimer disease) (T)	0.63	0.82	0.77	0.32	0.28	0.73
8	Los estudios de neuroimagenes (tomografía y/o resonancia magnética) pueden por sí solos decir si una persona tiene demencia frontotemporal (The results of a brain scan by itself can tell you whether a person has frontotemporal dementia) (F)	1.39	0.87	-0.85	0.69	0.3	0.73
9	La personas con demencia frontotemporal tienen mejor desempeño cuando deben elegir entre varias opciones predefinidas (People with frontotemporal dementia do best when given choices among many options) (F)	0.84	0.92	0.33	0.42	0.23	0.73
10	Existen tratamientos para disminuir la velocidad de progresión de la demencia frontotemporal (There are treatments to slow down frontotemporal dementia) (F)	0.76	0.87	0.49	0.38	0.38	0.72
11	Luego de que aparecen los primeros síntomas de demencia frontotemporal, la expectativa de vida media es de 7 a 13 años (After symptoms of frontotemporal dementi aappear, the average life expectancy is 7 to 13 years) (T)	1.09	0.85	-0.17	0.54	0.27	0.73
12	Basándose en la edad, es más probable que desarrollen demencia frontotemporal las personas que rondan los 70 años de edad en comparación con las personas que rondan los 50 años (On the basis of their age, people who are 70 years old are more likely to develop frontotemporal dementia than people who are 50 years old) (F)	1.05	0.91	-0.1	0.53	0.52	0.70
13	En línea general los cuidadores de personas con demencia frontotemporal reportan mayores niveles de estrés que los cuidadores con otras formas de demencia (On average, caregivers of people with frontotemporal dementia report more stress than caregivers of people with other dementias) (T)	1.43	0.75	-0.88	0.71	0.20	0.73
14	Las medicaciones diseñadas para mejorar la cognición y memoria en personas con Alzheimer son también apropiadas para personas con demencia frontotemporal (Medications designed to improve memory and thinking in people with Alzheimer disease are also appropriate for people with frontotemporal dementia) (F)	1.02	0.9	-0.04	0.51	0.40	0.72
15	Las variantes del lenguaje de la demencia frontotemporal son más comunes que la variante conductual (The language variant of frontotemporal dementia is more common than the behavioral variant) (F)	1.22	0.85	-0.43	0.61	0.43	0.71
16	Los pacientes con la variante conductual de la demencia frontotemporal suelen tener dificultad en evocar eventos del pasado (People with the behavioral variant of frontotemporal dementia have difficulty remembering events from the past (F)	1.19	0.89	-0.38	0.59	0.43	0.71
17	Las personas con la varianteconductual de la demencia frontotemporal en general carecen de interés en las cosas que antes disfrutaban (People with the behavioral variant of frontotemporal dementia lack interest in things they used to find enjoyable) (T)	1.58	0.66	-1.32	0.79	0.20	0.73
18	Las personas con las variantes del lenguaje de la demencia frontotemporal son capaces de leer y escribir sin dificultad (People with the language variant of frontotemporal dementia are able to read and write without difficulty) (F)	1.22	0.88	-0.46	0.61	0.32	0.72

F: false statement; T: true statement.

### Validation Sample

The final version of the Spanish FTDKS, along with a demographic questionnaire, was distributed in a Google Forms format among health professionals using snowball sampling techniques. The survey was distributed among colleagues using social networks and email, both directly and using professional groups from the leading Argentine societies of health professionals. In this way, 134 responses were obtained exclusively from health professionals (neurologists, psychiatrists, clinical psychologists, and neuropsychologists).

In addition to responses to the Spanish version of the FTDKS, demographic data was collected including: age, sex, education, professional discipline/specialty, years of experience, academical or research activities, health system where they work (public or private), practice settings, number of patients seen per month, self-reported knowledge of FTD (prior to answering the FTDKS), and clinical experience with dementia. 

Results were analyzed to obtain a global Cronbach’s α value. An isolated item analysis was performed to determine skewness, item difficulty, item discrimination, and global α if the item is deleted.

The *item difficulty* evaluates the proportion of respondents who answer an item correctly.

The *item discrimination* indicates how well an item discriminates respondents’ knowledge. A high discrimination index indicates that the item works differently between respondents with higher and lower scores, suggesting that the item identifies respondents with more or less knowledge

The statistical analysis was performed using R version 4.0.0 “Arbor day” and the Psych^15^, sjPlot^16^, and Table2 packages^17^.

## RESULTS

### Sample characteristics

One hundred thirty-four health professionals completed the Spanish version of the FTDKS. There were no reported difficulties in understanding the instructions or in completion of the scale.

The sample characteristics are shown in Table 2. The mean age was 42.9 years (range = 25-77 years). Most of the professionals were highly educated and trained, with 80 (59.7%) having finished at least the residency and 82 (61.2%) having 8 or more years of clinical experience. The main area of work was with outpatients (n = 113), and most of the sample worked in the private sector (n = 77). Regarding experience, many of the professionals (n=66) saw more than 100 patients per month. Of the sample, the majority were neurologists (n=51), followed by psychiatrists (n=50) and clinical psychologists (n=15). From the total sample, 73 (54.5%) reported having academic or research-related activities. In terms of self-reported knowledge of FTD, the majority of professionals reported knowing “something” about the disease (n = 81), whereas a smaller percentage reported knowing “a lot” (n = 22) and only 1 respondent considered themselves an “expert” (n = 1). 

**Table 2.** Demographics of the sample.

Characteristics	Total (n=100)	
Age (years)	Mean (SD)	42.9 (12.5)
Sex	Female	78%
Education level	Ph.D., Master or Fellowship	41 (30.59%)
	Residency (complete)	39 (29.10%)
	Residency (undergoing)	10 (7.46%)
	University degree	44 (32.83%)
Professional discipline/specialty	Clinical psychologist	15 (11.19%)
	Neurologist	51 (38.05%)
	Neuropsychologist	10 (7.46%)
	Psychiatry	50 (37.31%)
	Other	8 (5.97%)
Years of experience in healthcare	0-4 years	31 (23.13%)
	5-10 years	40 (29.85%)
	>10 years	63 (47.01%)
Academical/research activity	Yes	73 (54.47%)
Health system	Private	77 (57.46%)
Practice setting	Outpatient clinic	113 (84.32%)
	Inpatient clinic	5 (3.73%)
	Emergency service	7 (5.22%)
	Chronic inpatient institution	9 (6.71%)
Patients seen per month	1-99	68 (50.74%)
	100-199	47 (35.07%)
	More than 200	19 (14.17%)
Perceived knowledge of FTD	None	3 (2.23%)
	A little	27 (20.14%)
	Moderate	81 (60.44%)
	A lot	22 (16.41%)
	Expert	1 (0.74%)
Experience in dementia	No experience	16 (11.94%)
	Some experience	44 (32.83%)
	Moderate experience	58 (43.28%)
	A lot of experience	12 (8.95%)
	Extensive experience	4 (2.98%)
Experience in FTD	No experience	39 (29.10%)
	Some experience	61 (45.52%)
	Moderate experience	30 (22.38%)
	A lot of experience	2 (1.49%)
	Extensive experience	2 (1.49%)

SD: Standard deviation; FTD: Frontotemporal dementia.

### Psychometric properties of the Spanish FTDKS

The mean score for the Spanish FTDKS was 19.78 (range = 4-32, SD 6.38). Table 1 shows the mean score per question for each item in the scale, the standard deviation (SD), the item difficulty, the item discrimination, and internal consistency (Cronbach α). The mean score per response ranged from 0.31 for statement 3 (“Among all people with dementia, 5-10% of them have frontotemporal dementia”, False) to 1.66 for statement 1 (“Frontotemporal dementia is a type of Alzheimer disease”, False). The internal consistency reliability (Cronbach α) for this sample was 0.74, 95% CI [0.67 ,0.8]), indicating acceptable reliability.

## DISCUSSION

The aim of the current study was to translate and adapt the original English version of the FTDKS published by Wynn et al. into Spanish. The results obtained show that the Spanish version has a good reliability and internal consistency and that it can be a useful tool to evaluate the knowledge of health professionals in the field of FTD.

A surprising result is the low mean accuracy score obtained for the third item (related to the prevalence of FTD). Considering the lack of a registry of FTD cases in Argentina, there are at least two interpretations for this. First, it is possible that the prevalence of the disease in our country is, in fact, lower than that reported in the international literature (from which the validity of the scale question is based). If this were the case, the answers given by the respondents would not be incorrect. However, according to the hypothesis that led us to start this work, it is possible that the information on prevalence is ignored or unknown by respondents and the low mean score on that item reflects a general lack of awareness of the disease by Argentine health professionals.

Another interesting factor is the low overall result obtained in the FTDKS by our sample. With a mean of 19.78 (SD 6.3) and a range of 4 to 32, out of a maximum of 36 possible points, these results are significantly lower than those described in the original article by Wynn et al. In that study, health professionals mean score on the FTDKS was 25 (SD = 5.47, range = 10 - 36). These results reinforce the notion that FTD is a poorly known illness among health professionals. With this instrument validated in Spanish, we propose to study the level of knowledge of professionals in Argentina and eventually throughout Latin America, focusing on the specialties that are likely to deal first with these patients due to the characteristics of the disease: neurologists, psychiatrists, clinical psychologists, and neuropsychologists.
